# Editorial: Embodied interfaces: human experience in virtual and mediated worlds

**DOI:** 10.3389/fnsys.2026.1911204

**Published:** 2026-07-06

**Authors:** Sergio Frumento, Danilo Menicucci, Simone Grassini, Valentina Cesari

**Affiliations:** 1 Department of Information Engineering, University of Pisa, Pisa, Italy; 2 Department of Surgical, Medical and Molecular Pathology and Critical Care Medicine, University of Pisa, Pisa, Italy; 3 Department of Psychosocial Science, University of Bergen, Bergen, Norway; 4 Social Cognition in Human-Robot Interaction, Italian Institute of Technology, Genova, Italy

**Keywords:** embodiment, immersive environments, mediated interaction, technology, telepresence

We no longer simply interact with interfaces. We interact through them, alongside them, and sometimes even as extensions of ourselves. This radical shift captures one of the most significant transformations in contemporary human-technology interaction: interfaces are no longer passive or screen-bound tools designed merely to optimize performance or efficiency (Dourish, 2001; Slater, 2009). Virtual reality (VR), augmented reality (AR), telepresence systems, robotic platforms, and immersive digital environments are becoming progressively embodied and situated systems that reshape perception, cognition, and bodily self-awareness. In this rapidly evolving scenario, the critical challenge is not only technological but also profoundly human: understanding what happens to the individual within technologically mediated interactions.

This Research Topic, *Embodied interfaces: human experience in virtual and mediated worlds*, was conceived to contribute to this growing interdisciplinary dialogue by bringing together experimental, theoretical, and review-based contributions spanning cognitive neuroscience, psychology, and human-computer interaction.

A pivotal theme emerging from the collected articles is the plasticity of bodily self-representation in immersive environments, promoting the malleability of human body boundaries.

Several contributions explored how virtual embodiment, avatar characteristics, and mediated perspectives can modulate ownership, agency, and the sense of presence. Hartfill et al. found that self-configured VR avatars were associated with higher attractiveness and more positive valence ratings, as well as a stronger subjective response to a virtual threat directed toward the avatar; however, explicit embodiment measures remained inconclusive. Wright et al. advanced a theoretical framework that may explain why embodiment may rely less on anatomical realism (physical appearance) and more on whether VR preserves perception, recognition, action, and feedback. Elovainio et al. extended this to social VR, showing that feeling a virtual space as collectively “ours” is associated with lower explicit prejudice. Kapah and Freedman focused on adolescents, emphasizing the psychological relevance of immersive self-representation during sensitive developmental stages. Their findings emphasize the important role of virtual mirrors and embodiment in enhancing users' sense of presence and suggest that these mechanisms may contribute to short-term improvements in self-esteem.

Taken together, these studies suggest that the psychological efficacy of VR is not merely ascribable to its realism but rather to the creation of meaningful loops between the body, the self, other individuals, and the environment. The efficacy of embodiment is contingent upon the ability of users to act, perceive feedback, identify with the avatar or space, and experience the virtual world as personally or socially relevant.

Another core dimension of this Research Topic is the growing integration of neurophysiological and affective dimensions into human-interface interaction. In addition to behavioral performance and subjective self-report measures, several studies emphasized the burgeoning need to identify physiological markers associated with embodiment, emotional engagement, and immersive processing during interaction with interfaces. Esteves and Vourvopoulos conducted a systematic review of EEG biomarkers associated with the sense of embodiment, identifying reduced alpha-band activity over central-parietal regions as the most recurrent neural correlate of embodiment. However, the study did not identify an EEG signature that reached sufficient consistency to serve as an embodiment biomarker. The authors emphasized the need for standardized protocols to develop reliable neural markers of embodiment for use in virtual reality (VR) rehabilitation, brain-computer interfaces (BCIs), and clinical neuroscience.

Complementing this perspective, Thomas et al. reviewed physiological responses to sensory stimulation in immersive environments, highlighting that virtual sensory stimuli reliably trigger autonomic physiological responses even without physical counterparts. Across studies, emotional and thermal virtual stimuli were associated with changes in heart rate, galvanic skin response, and heart rate variability, supporting the idea that immersive reality can induce genuine bodily reactions with potential applications in clinical fields. Additional contributions explored the emotional and neurobiological correlates of the immersive experience, including the neural basis of awe-related processes in affective disorders investigated by Bondi et al. In this study, the authors highlighted that awe-inducing VR modulates theta- and alpha-band activity differently in healthy and clinical populations. Participants with affective disorders showed altered neural integration and reduced emotional differentiation.

Collectively, these studies reflect a broader shift within the field toward understanding immersive technologies as fundamentally embodied and neurocognitive experiences that engage perceptual, affective, and physiological systems simultaneously.

The articles included in this Research Topic also underscore the importance of individual variability in technologically mediated interaction. Psychological traits, cognitive styles, prior expertise, attentional strategies, and embodied interaction processes appear to shape how individuals perceive, navigate, and act within virtual environments. Cesari et al. introduced the FOUND questionnaire as a tool for identifying stable traits associated with successful remote operations, thereby highlighting the relevance of psychological profiling in technologically mediated contexts. Similarly, Koshizawa et al. demonstrated that competitive players exhibit earlier and more accurate predictive saccades during target-tracking tasks compared to non-competitive players, with this advantage being associated with increased low-beta EEG activity in brain regions involved in visuomotor integration, spatial prediction, and motor planning. Kuratomo et al. explored the honey-pot effect on pedestrian attention to public displays in virtual environments, emphasizing the ecological and social dimensions of mediated interaction. Finally, Rennó-Costa et al. advanced a framework for immersive creativity and expressive interaction in VR and mixed reality, emphasizing embodied interaction, navigable creative spaces, constrained freedom, and collaborative world-building as key principles for designing immersive systems that enhance human creativity and expression.

Taken together, these contributions suggest that universal models of interaction alone are insufficient, and that increasingly adaptive, personalized, and human-centered approaches are needed to account for interindividual differences and contextual variability.

**Figure 1:**
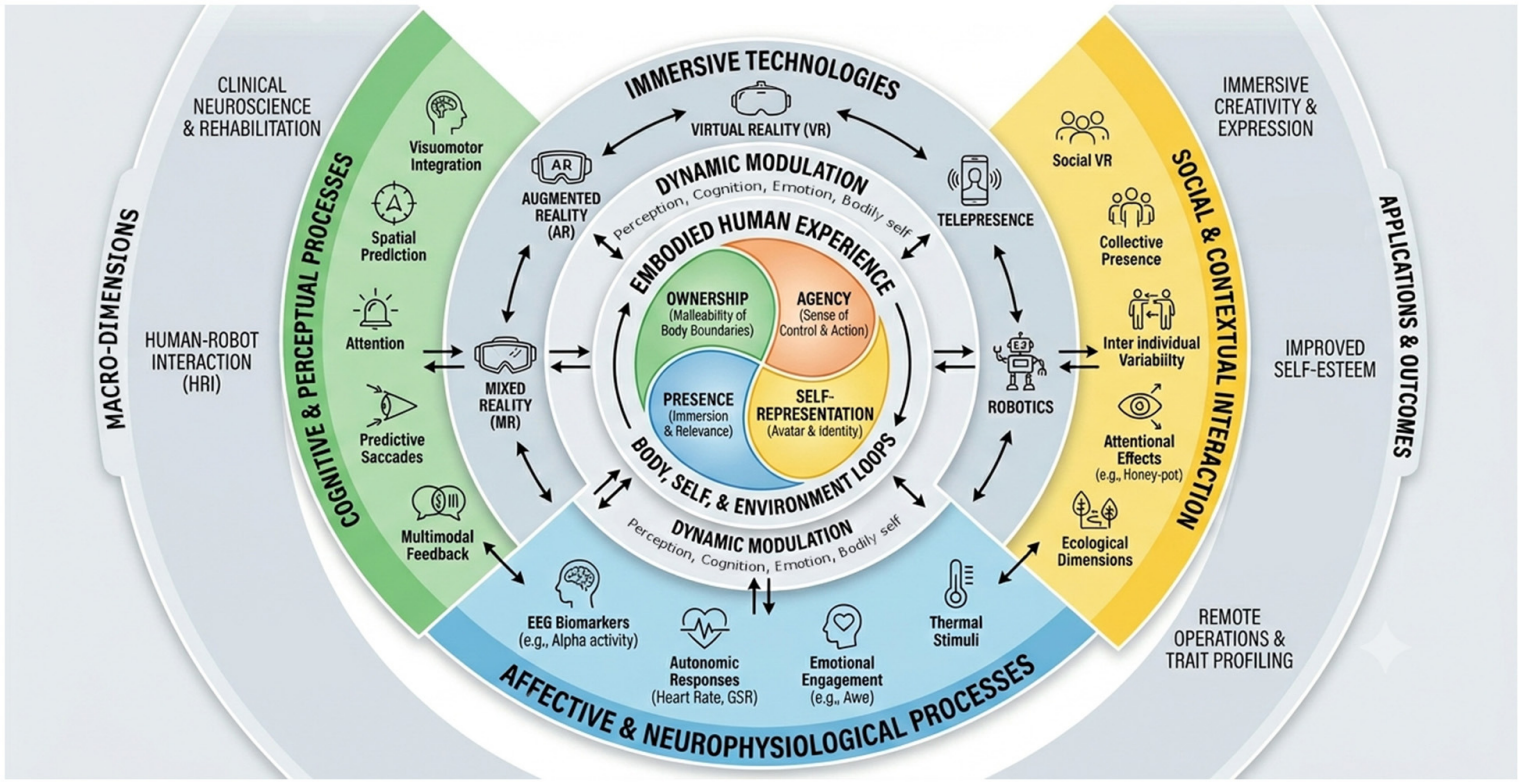
Integrative framework of embodied interfaces and technologically mediated human experience. A proposed multilayer model of embodiment in immersive and mediated environments, in which ownership, agency, presence, and self-representation are core components of the embodied human experience. These dimensions emerge through dynamic interactions between the body, the self, and the environment, and are continuously modulated by perceptual, cognitive, emotional, and neurophysiological processes. The surrounding layers highlight the role of mediated technologies, comprising virtual reality (VR), augmented reality (AR), mixed reality (MR), telepresence, and robotics, in shaping the human experience via cognitive-perceptual mechanisms, affective and physiological responses, and social-contextual interactions. The outermost layer emphasizes the potential application domains and outcomes of this framework, ranging from clinical rehabilitation and human–robot interaction to remote operations, social VR, creativity, and psychological well-being, underscoring the need for an integrative and human-centered approach to the study of embodied technologies.

In summary, this Research Topic highlights the complex, multidimensional nature of technologically mediated human experience and the urgent need for integrative frameworks that encompass neuroscience, psychology, embodied cognition, and human-computer interaction research (Figure 1). Future developments in the field will likely benefit from multimodal and longitudinal approaches that combine neurophysiological measures, behavioral indices, and first-person subjective reports (preferably continuous rather than punctual pre-/post- evaluations) within ecologically valid immersive paradigms. As immersive technologies continue to evolve, their significance will depend not only on increasing realism and interactivity but also on our ability to understand how these systems actively shape perception, cognition, emotion, and the embodied sense of self (Slater and Sanchez-Vives, 2016). Ultimately, the challenge lies not simply in the technology itself, but also in understanding the human being within increasingly mediated realities.
